# Effects of mechanical ventilation on neurodevelopment at 12 months in preterm low birth weight pediatric patients: a systematic review

**DOI:** 10.3389/fped.2024.1363472

**Published:** 2024-10-21

**Authors:** Valerie Vargas Caicedo, Marta de la Plaza San Frutos, Maria Dolores Sosa Reina, Maria Garcia Arrabe, Federico Salniccia, Clara Reina Aguilar, Cecilia Estrada Barranco

**Affiliations:** ^1^ Universidad Europea de Madrid, Faculty of Sport Sciences, Villaviciosa de Odón, Spain; ^2^ Servicio Medicina Intensiva, Hospital de Mataró, Barcelona, Spain

**Keywords:** mechanical ventilation, newborn, neurodevelopment, premature birth, invasive mechanical ventilation, non-invasive ventilatory support

## Abstract

**Introduction:**

The objective of this review is to know the existing scientific evidence about the effects of mechanical ventilation (MV) on neurological development in low-birth-weight premature pediatric patients after 12 months of life, taking as background the direct impact that ventilation has on the central nervous system in the newborn during the first days of life.

**Methods:**

A systematic search was carried out between 2003 and 2024 in the data bases of: PUBMED, Cochrane Library Plus, PEDro, CINAHL, and SciELO, and two investigators scored the articles according to the Newcastle-Ottawa Assessment scale.

**Results:**

Were found 129 non-replicated articles, and 10 cohort and cross-sectional studies were selected that performed an assessment of neurodevelopment in the three spheres after 12 months of life in corrected age of premature infants exposed to ventilator support and related the two variables independently.

**Conclusions:**

Mechanical ventilation is an independent neurodevelopmental risk factor in low-birth-weight preterm infants. The time of exposure and the type of ventilation were the variables with the most scientific evidence.

**Systematic Review Registration:**

https://www.crd.york.ac.uk/, Identifier CRD42023446797.

## 1 Introduction

Newborns born before 37 weeks of gestation are considered a premature population (1). According to the WHO, prematurity is one of the most significant risk factors for postnatal complications and development (1), because there is a greater probability of presenting surfactant deficiency due to lack of pulmonary maturation, which hinders gas exchange (2), weakness of the respiratory musculature, a more distensible rib cage as a result of the maturational deficit, and an irregular respiratory rhythm that leads to ventilatory failure (3); factors such as: hyaline membrane disease, meconium aspiration syndrome, congenital sepsis, ischemic hypoxia, and prenatal brain injuries (among others) (4), this might mean the need to initiate mechanical ventilation (MV) as life support in the first hours of the life of the premature infant. This intervention will vary depending on the infant's clinical condition.

There are two types of mechanical ventilation: invasive (IMV) and noninvasive (NIV). IMV requires endotracheal intubation through the nose or mouth (3). Three general modes of IMV are programmed according to the needs of the neonate: controlled, assisted-controlled, and spontaneous (5). NIV is the support provided without invading the airway; pressure chambers, facial/nasal masks, or high-flow cannulas are used for this type of ventilation, with two modalities: extrathoracic negative pressure or inspiratory positive pressure systems (5).

In neonates, NIV intervention has increased in delivery rooms by 47.8% with modalities such as continuous positive pressure, reducing endotracheal ventilation interventions from 6% to 4% (6).

Authors such as Van Kaam et al. (5) and López et al. (7) have published publications that explain the benefits and consequences of the choice between invasive and noninvasive ventilation; however, all three conclude that the medical team's decision to connect a patient to one support system or another should be based on the evaluation of the functioning of all systems and the patient's global condition.

MV directly impacts all the newborn's systems, mainly the heart, lungs, and brain relationship in the first days of life (2). There is an interdependence of the support and the different systems that have not matured, causing alterations in the infant's development, and it has been demonstrated that it is a high-risk factor for neurological development (8). Morbidities such as moderate sensory, motor, and severe hearing impairment are directly related to both types of ventilatory support (9, 10).

Brain lesions and developmental delay due to ventilation are due to two main causes: migration to the brain of the pulmonary inflammatory cascade, causing a focal lesion that increases markers of oxidative stress (overproduction of cytokines) and consequently injury to the white matter (11); and the second is hemodynamic instability, since the over distension of the alveoli causes compression of the capillaries, increasing pulmonary resistance and decreasing cardiac output, which leads to variable blood flow to the brain and extravasation of brain proteins (12).

Research has shown that early stimulation by the interdisciplinary therapy team benefits the development of the neonate in the critical care unit since the recognition of his environment facilitates the generation of motor action in accordance with his corrected age (13, 14).

Knowing the impact of ventilatory support on the neurological system would allow us to provide more precise information to develop personalized strategies that respond to the needs of each patient. This would reduce or prevent possible sequelae in the neurodevelopment of the premature infant, thus improving the quality of the service provided by physiotherapy professionals.

## 2 Materials and methods

### 2.1 Study selection

A systematic review of the studies published on the influence of mechanical ventilation on neurological development in preterm patients between 2003 and August 2024 was carried out, following the PRISMA (Preferred Reporting Items for Systematic Reviews and Meta-Analyses) checklist (15).

The protocol was registered in the International Prospective Register of Systematic Reviews (PROSPERO, CRD42023446797).

The literature search was conducted by two independent investigators (VV) (VB) independently, in the following databases: PUBMED, Cochrane Library Plus, PEDro, CINAHL and SciELO, following the PICO question to organize and analyze the information found, which is: How do mechanical ventilation and its variables positively or negatively influence neurological development in preterm pediatric patients with low birth weight after 12 months of life?

The search terms used for this review were “mechanical ventilation” AND (“neurological development” OR “motor development” OR “development”) in “premature”; however, when starting the search, the population filter was performed with the term “neonates” in the PUBMED and PEDro databases, since some literature uses different terminology to refer to the same population.

The inclusion criteria taken into account were:

• Analytical cohort studies (retrospective and prospective) and descriptive cross-sectional studies.
• Articles where the study population was preterm infants requiring mechanical ventilation.
• Articles in English and Spanish.
• Articles published between 2003 and August 2024, considering advancements in technology and updates to clinical practice guidelines up to this date (16, 17).
• Articles directly correlating mechanical ventilation with motor development.
And the exclusion criteria:
• Articles that within their methodology performed a direct intervention to the study population by the investigators.
• Retrospective articles of a single case.
• Articles that include in the neurodevelopmental analysis a population under 12 months of corrected age.

### 2.2 Classification of studies for analysis

Based on the established search criteria, studies were grouped according to the correlation between mechanical ventilation and neurodevelopment, focusing on the following variables: mechanical ventilation as a risk factor, duration of mechanical ventilation (exposure time), type of mechanical ventilation (invasive and noninvasive), and specific variables within invasive mechanical ventilation (such as ventilatory mode).

### 2.3 Evaluation of the methodological quality of the studies

Two authors independently performed data extraction using a standardized data collection notebook, where the following data were considered: type of study, age of participants, correlation, and time elapsed between the variables studied (mechanical ventilation and neurodevelopment). The methodological quality of the articles included in this study was assessed using the Newcastle-Ottawa Assessment Scale for observational studies (18), which evaluates 8 items: patient selection (4 items), comparability (1 item), and study outcomes (3 items).

Each item allows for multiple selections, but points are only awarded to those that meet the criteria marked with an asterisk in the instrument, with a maximum possible score of 9 and a minimum of 0, as up to 2 points can be awarded for the comparability item.

To classify the quality of the studies, it is established that: a score of 80% or higher indicates a high-quality study; a score between 70% and 80% indicates medium quality; and studies scoring less than 70% are considered low quality (18).

## 3 Results

### 3.1 Selection of studies

**Figure 1:**
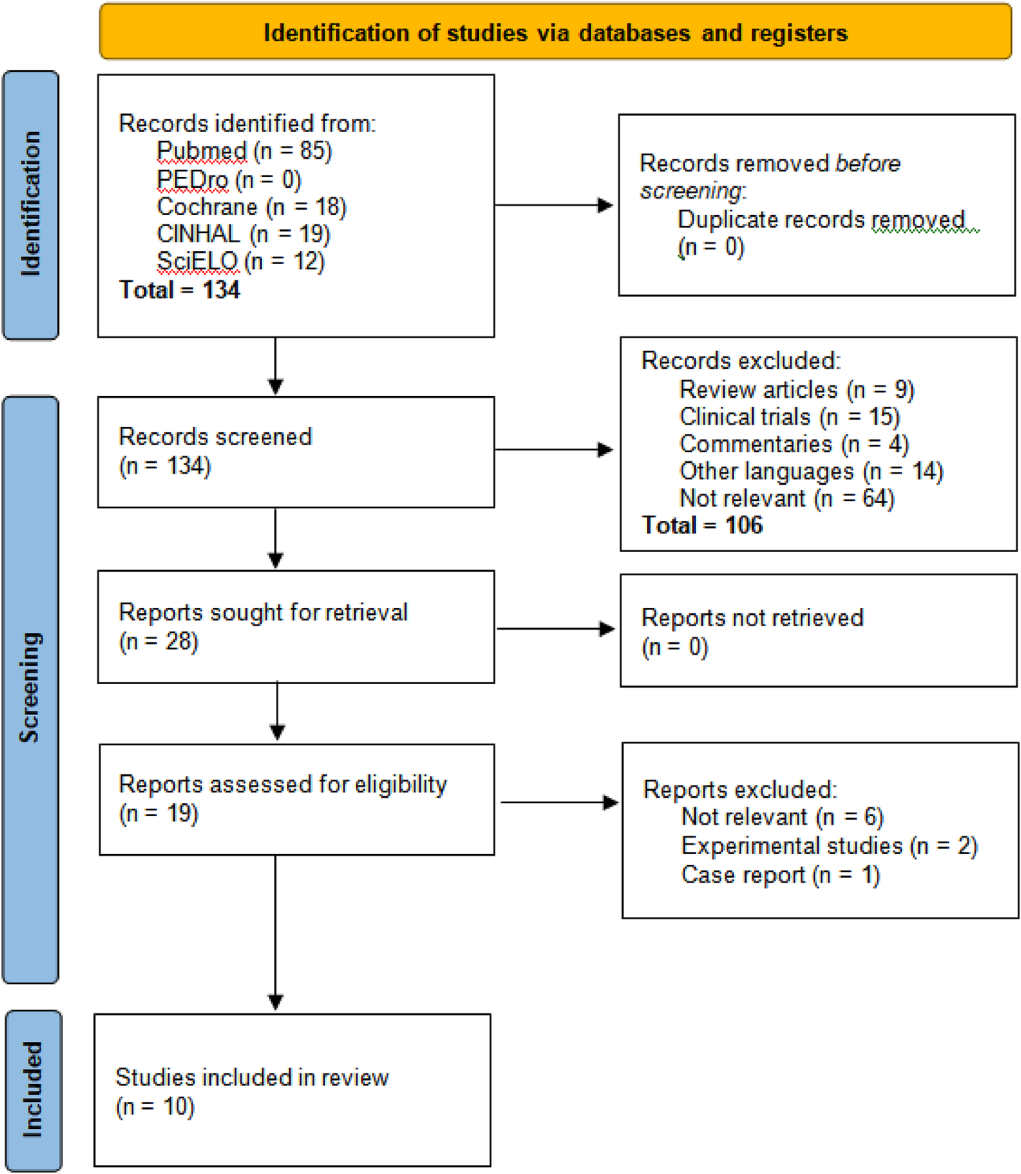
Selection flow diagram.

When we applied the search strategies in the five databases, we initially obtained 134 results as follows: PUBMED 85, PEDro 0, COCHRANE 18, CINAHL 19, and SciELO 12. After reviewing the titles and abstracts, 106 articles were excluded in the first stage due to the following reasons: 9 were systematic reviews of mechanical ventilation (MV) in premature neonates and its causes, 15 were clinical trials, 4 were commentaries on how the literature predictively supports the effect of MV in premature neonates, 14 were in a language other than English or Spanish, and finally, 64 were unrelated articles (Figure 1).

Subsequently, a thorough reading of the 19 preselected articles was performed, leading to a second selection phase where 9 articles were discarded based on the exclusion criteria: six (6) did not present a relationship between mechanical ventilation (MV) and neurodevelopment, two (2) involved two-part analysis studies where the first part was an experimental trial, and one (1) focused on a single case.

As a result, ten (10) observational studies were finally selected for inclusion in the present study. The second evaluator (VB) reviewed the selection and found no discrepancies in the choice of articles (Table 1).

**Table 1:** Characteristics of observational studies.

Study (reference)		Subjects (N, gestational age and corrected age, weight) MV	Exposure (Duration, type of MV and variables in MV)	Outcome measurements	Outcome measurements (Relationship between MV and neurodevelopment)
1	Guillot et al. (10) Duration of MV and neurological development.	N: 144 children included - 117 children in follow-up (82%). GA: less than 30 GS. (M: 27.1) P: Less than 2,000 g EC: 4 years	Time and type of MV	Movement Assessment Battery for Children 2nd Edition (M-ABC 2) Wechsler Primary and Preschool Intelligence Scale 4th edition.	Prolonged ventilation in neonates was associated with a decrease of 4.6 points/10 days of MV on the M-ABC2 and no relationship was found between the duration of NIV and the impact on motor performance at preschool age.
2	Asztalos et al. (23) Factors associated with neurodevelopment	N: 2,069 children included - 1,660 children in follow up (80.2%) GA: younger than 29 GS. CD: 18–21 months.	Time on positive pressure mechanical ventilation.	Bayley III Scale	The reduction of time on positive pressure per week accompanied by a decrease in parenteral nutritional support was associated with a 2% to 10% increase in the score in the three areas of the Bayley scale.
3	Velikos et al. (19) Relationship of neurodevelopment and environmental factors	N: 120 children included and in follow-up (100%). GD: Less than 32 SG. (M: 28.6) P: 590–2,000 g (M: 1,178) EC: 12 months.	Time on high-frequency and invasive pressure ventilation within 32 weeks after birth (n.58).	Bayley III Scale	In the bivariate analysis of sociodemographic factors, they determined that male neonates who required transfusions and were exposed to prolonged MV obtained lower scores in the areas of cognition, language and motor skills, with a mean score below the 50th percentile.
4	Bulbul et al. (20) Motor development in preterm infants	N: 96 children included and in follow-up (100%). GA: Less than 34 GS. (M: 30.5) P: 625–2,900 g (M: 1,542) EC: 18–24 months.	Time on mechanical ventilation (n.42) and CPAP (n.68)/day	Bayley III Scale	Neonates with prolonged mechanical ventilation presented low scores in the three areas, with the motor area showing the greatest statistical deficiency, with a relationship between MV support with Apgar scores below 7 and the need for resuscitation in the first 24 h of life.
5	Guangxi et al. (26) Neurodevelopment in preterm infants	N: 159 infants included and 131 in follow-up (82.3%). GA: less than 28 GS. P: 1,000–1,500 g EC: Between 18 and 24 months.	Time and exposure in mechanical ventilation (CPAP and invasive)	Gesell Development Scale Developmental quotient	They concluded that the duration of MV is a determining factor for neurological development in a negative way, taking into account that MV longer than 5 days is already considered prolonged.
6	Saldir et al. (24) Neurodevelopment in infancy	N: 188 children included and 169 in follow-up (89.8%). GA: Between 24 and 32 SG. (M:30.1) P: Less than 1,500 g They were assessed 12 months after birth.	Time and type of mechanical ventilation (intermittent mandatory and high flow nasal cannula pressure).	Bayley III Scale	The duration of mechanical ventilation longer than 21 days in neonates showed a low score on the Bayley Scale, associated with abnormal neurological development. They also showed that the time in NIV was shorter than in IMV.
7	Guerra et al. (21) Delays in low birth weight preterm infants.	N: 122 children included and 100 in follow-up (81.9%). EG: Less than 36 GS. P: Between 1,500 and 1,999 g EC: Between 18 and 24 months.	Mechanical ventilation as an independent risk factor.	Bayley III Scale	MV as an independent factor had a greater impact on the cognitive area with a decrease of 6 points below the mean; it was also associated with an increased risk of venous pulmonary hypertension, a determining factor in delayed motor development.
8	Nazi and Aliabadi (22) Motor development with and without mechanical ventilation	N: 110 children included and in follow-up (100%). EG: Less than 34 SG. (M:30.4) P: Less than 2,500 g (M: 1,410 g) EC: Between 8 and 12 months.	MV exposure (n.35) with respect to preterm infants without MV (n.35) and term neonates (n.40).	Peabody Developmental Motor Scale 2	In fine motor skills, there were differences between the group of term and preterm infants; however, within the preterm group that required IMV and those that did not, there were no major differences; but in gross motor skills, those that did require IMV obtained lower scores with a mean deviation significantly lower than the average.
9	Thomas et al. (25) Neurodevelopment according to type of ventilation.	N: 307 children included and 208 in follow-up (67.7%). EG: Less than 30 SG. P: Less than 1,000 g. EC: Between 18 and 22 months.	Comparing IMV and NIMV (CPAP)	Bayley III Scale	The delay in development presented by those in the IMV group compared to the NIMV group is directly associated with the ventilatory type, obtaining a score below the mean and a higher probability of death in the first 32 weeks.
10	Stefanescu et al. (27) Neurodevelopment according to ventilatory mode.	N: 270 children included and 155 in follow-up (57.4%). EG: Between 22 and 32 SG. P: Less than 1,250 g EC: 18 months.	Compare invasive mechanical ventilation mode (PCV and VGPSV).	Bayley III Scale	Neonates with VGPSV ventilation presented fewer complications in the unit and shorter duration of ventilation than those in the PCV group; and although there was a higher score in the VGPSV group in the three developmental areas it is not statistically significant compared to the PCV group.

IMV, invasive mechanical ventilation; PCV, pressure controlled ventilation; PSVPV, pressure support ventilation with volume guarantee.

### 3.2 Methodological quality of studies

The Newcastle-Ottawa Assessment Scale was used to evaluate the methodological quality of the articles (Table 2). The 10 studies scored between 7 and 8; 8 articles received a high methodological rating, and 2 were rated as medium quality. It is important to note that 4 of the articles with high ratings did not apply the cohort sample item since they are cross-sectional control studies. Out of the 90 items assessed, there was a discrepancy in only one, which was resolved by consensus between the two evaluators (VV and VB).

**Table 2:** Newcastle-Ottawa assessment scale in cohort and cross-sectional studies.

Quality assessment	Acceptable criteria	Saldir et al. (24)	Thomas et al. (25)	Guerra et al. (21)	Velikos et al. (19)	Nazi and Aliabadi (22)	Stefanescu et al. (27)	Asztalos et al. (23)	Guangxi et al. (26)	Guillot et al. (10)	Bulbul et al. (20)
Exposed cohort representative?	Representative of average community?	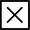	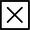	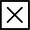	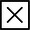	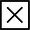	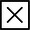	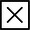	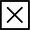	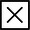	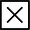
Selection of non-exposed cohort?	Drawn from same sample as exposed cohort?	N.A.	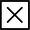	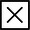	N.A.	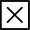	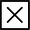	N.A.	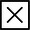	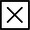	N.A.
Ascertainment of exposure?	Structured interview?	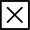	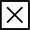	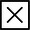	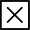	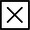	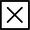	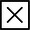	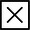	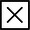	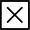
Outcome at baseline?	Incidence of neurological disease?	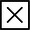	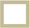	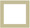	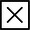	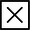	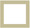	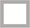	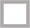	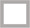	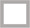
Controls for important factors?	Adjusted for age and weight?	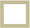	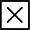	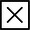	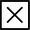	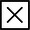	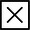	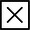	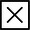	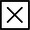	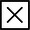
Controls for additional factors?	Adjusted for at least 2 other (risk) factors?	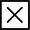	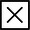	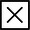	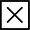	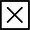	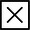	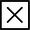	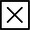	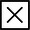	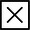
Assessment of outcome?	Assessed through the developmental scale?	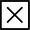	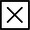	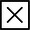	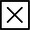	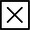	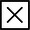	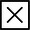	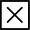	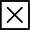	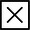
Adequacy of follow-up duration?	Follow-up duration ≥12 months?	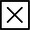	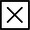	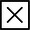	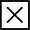	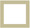	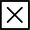	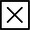	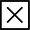	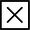	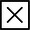
Adequacy of lost at follow-up?	Complete follow up? Bias unlikely through lost cases?	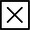	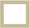	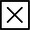	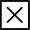	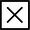	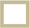	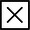	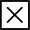	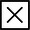	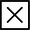
Score:		7 High	7 Medium	8 High	8 High	8 High	7 Medium	7 High	8 High	8 High	7 High

### 3.3 Study characteristics

Of the studies, 4 (19–22) included preterm infants (less than 36 weeks of gestation), 4 others (10, 23–25) included very preterm infants (less than 32 weeks of gestation), and 2 studies (26, 27) included infants ranging from very preterm to extremely preterm (less than 28 weeks of gestation). All the studies are heterogeneous because the patients exposed to mechanical ventilation showed variations in the mode, type, and duration of ventilation, as there is no universal protocol for invasive methods, which varies according to the patient's clinical condition.

In the 10 articles, a total of 2,866 cases were followed up, with a mean of 286.6 ± 483.805; the range of gestational age was between 24 and 36 weeks, and birth weight ranged from 590 to 2,500 kg. Regarding data collection and analysis of the results, all the articles used neurodevelopmental assessment scales in the three domains (cognitive, language, and motor), providing a final follow-up for the patients who continued in the study. It is worth noting that only one article did not mention the average corrected age of the evaluated patients (24), and two articles had a high probability of bias due to a loss to follow-up of more than 20% of the total cases (25, 27).

### 3.4 Influence of mechanical ventilation on neurodevelopment

Regarding the factors of mechanical ventilation that were related to the influence on neurodevelopment in the different studies of this review, the results were analyzed and categorized as follows.

#### 3.4.1 Duration of mechanical ventilation

Among the 10 articles reviewed, 6 investigated the duration of mechanical ventilation (MV) as a risk factor (10, 19, 20, 23, 24, 26). Five of these studies found that longer MV duration was associated with a higher likelihood of delayed motor development. The studies used different criteria to define prolonged MV: Guangxi (26) considered MV prolonged if it lasted more than 5 days, Saldir et al. (24) defined it as more than 21 days, and three other studies identified increased risk if MV extended beyond 7 days (15, 18, 21). In contrast, one study did not find a direct relationship between MV duration and delayed motor development (10), although this study involved children receiving non-invasive ventilation (NIV).

On the other hand, three articles considered factors that prolonged MV exposure, being an influential component at the moment of relating time with the consequences on development (19, 20, 23).

Velikos et al. (19) presented in their conclusions that there are greater alterations in the areas of cognition and language development, while Bulbul, et al. (20) found that the delay is greater in the motor area; and authors such as Asztalos, et al. (23) and Saldir et al. (24) determined that there is a global neurological development type, without finding significant differences between the three spheres of development.

#### 3.4.2 Type of mechanical ventilation

Two articles studied the relationship of IMV and NIV as a risk factor for neurodevelopment (23, 25). Between these two was included an article already mentioned in the category of duration in MV (23).

Both studies agree that in NIV there is a lower probability of presenting neurodevelopmental delay. One of the two articles evaluated a population with more homogeneous characteristics and exposed to similar risk factors. Therefore, in its conclusion, they mention a direct relationship between NIV and delay in the three developmental spheres (25).

In addition, another article previously mentioned in the section on duration, in its discussion, it mentions that weaning from ventilation in neonates who were exposed to noninvasive measures was performed in less time compared to those of IMV, presenting better results (24).

#### 3.4.3 Mechanical ventilation as a risk factor

Two articles studied MV independently as a risk factor for neurodevelopment. Both conclude that MV has a great impact on developmental delay, with the motor area showing the greatest deviation from the mean in the rating scales (21, 22); one of the articles presented more specific results, demonstrating that IMV has a greater impact on fine motor skills compared to gross motor skills in the short term in the child's motor development (22).

#### 3.4.4 Mechanical ventilation mode

Only one article studied the IMV mode (27), where they demonstrated that, among the pressure ventilatory mode compared to a volume mode, the latter has a better impact on neurodevelopment since patients who were ventilated with this mode required less ventilation time and the results within the rating scale were not far from the mean, however due to the number of population and the little research there is about this topic the results are not statistically significant.

## 4 Discussion

This systematic review of observational articles aims to know the influence of mechanical ventilation and its variables on motor development in premature infants after 12 months of life (no-corrected age).

During the search we did not find in any database, reviews that related mechanical ventilation with neurodevelopment in the pediatric population (independently of gestational age). One review presented the effects of permissive hypercapnia during MV on neurodevelopment based on the alterations that can occur in the nervous system; however, they do not mention if there was a follow-up of preterm infants or if they applied neurological evaluation scales in the studies they included (28). Another review presented neurodevelopmental delay as side effects in only one ventilation modality: continuous positive airway pressure (CPAP), however, the limitations specified that only one of the comparison studies reported these results (29). In one review they studied the effects of different interfaces in NIV (30) and another one, the efficacy of different modalities in noninvasive support (31), however, they did not conclude whether they had a direct effect on neurological development, since none of the articles they included reported it. While our inclusion criteria covered studies from 2003 to August 2024, only those published between 2010 and August 2024 ultimately met these criteria, reflecting the alignment with recent advancements and evolving practices in neonatal care.

All the studies included in this review used various tools to measure neurodevelopment. Despite the variability in these tools, they are consistent in their core objective—assessing child development. Although the methodologies of the neurodevelopmental assessment tools differ, they all aim to evaluate the same fundamental concept of developmental progress. All the studies concluded that mechanical ventilation negatively impacts one or more aspects of neurodevelopment, regardless of differences in ventilation duration, type, or mode. However, in the study carried out by Saldir et al. (24) they did not find a direct relationship between alterations in development and non-invasive mechanical ventilation, possibly because this measure was taken as the main life support strategy in the first 24 h of the premature infant.

The duration of mechanical ventilation is the variable with the strongest evidence in this review. According to the included studies, prolonged ventilation is directly related to the alterations that preterm infants presented in their neurological development, because in the articles the ventilation variable was studied independently of the health conditions of the preterm infants (10, 19, 20, 23, 24, 26). The results of the scales applied in each study recorded a statistically significant deviation from typical development in premature infants who required long-term ventilatory support, so that the prognosis presented in the population under study was a delay or abnormal neurological development; even so, there is no clear consensus on how long is considered “prolonged mechanical ventilation”, with a great variety in the number of days taken as reference (from five to twenty-eight). Authors such as Pierrat et al. (32) corroborate that early weaning from ventilation decreases the probability of presenting alterations during neurological development in the first two years of life.

NIV has had a more favorable score in comparison to IMV in the neurodevelopment of premature infants and has proven to be a beneficial measure, decreasing clinical complications during exposure to ventilatory support (23, 25); likewise, the authors concluded that ventilation is an independent factor to the comorbidities of neonates, therefore, the use of less invasive measures such as continuous positive airway pressure (CPAP) and high frequency oscillatory ventilation is a factor that positively influences neonates. Onland, et al. (33) concluded that the use of less invasive measures in conjunction with other adjuvant treatments decreases the risk of developmental delay and lower probability of mortality in extremely preterm infants. However, recent studies cast doubt on this conclusion, highlighting that more research is needed to determine the failure or success of one or the other ventilatory support, as complications during pregnancy or the first 72 h of the preterm infant's life have been shown to be key factors in the medical team's decision-making process (34).

Factors such as the programming of ventilatory parameters are adjusted according to the needs and evaluation of the patients (7), therefore, the ventilatory mode is a variable dependent on the health status of each premature infant. Research has shown that volume-driven ventilatory modes minimize the risks of morbidity and mortality compared to pressure-driven modes but are not a factor influencing neurodevelopment (7, 27). Recent reviews evaluated whether permissive carbon dioxide (CO2) levels during ventilation affected different systems via respiratory rate programming, concluding that both hypercapnia (<35.3 mmHg) and hypocapnia (>30.3 mmHg) are strategies with variable and therefore contradictory results, which did not allow us to determine whether or not they influence the neurological development of the premature infant; in addition, the reference values of CO2 are subject to the weight of the neonate, so that the heterogeneity of the participants was another limitation to establish a definitive result (35).

The need for mechanical ventilation in the neonate has become a subject of study of great importance due to the implications of the use of this vital support and its relationship with neurodevelopment, which is not only altered by direct exposure, but also by associated clinical factors such as: pulmonary dysplasia, infections, pulmonary hypertension, damage to the auditory canal, damage to the white matter, among others; which implies more time in the critical care unit and longer duration of ventilation, thus increasing the probability of presenting developmental delay (36).Therefore, studies such as that of Thomas et al. (24) and Saldir et al. (25) included in their study population neonates with greater homogeneity concerning the risk factors to which they were exposed and in the studies of Velikos et al. (19) and Asztalos, et al. (23) the clinical characteristics of each of the study participants were studied independently to determine the direct impact of MV on neurodevelopment.

Knowing the role of the physical therapist in the critical care unit in premature patients with ventilatory support and the impact of early interventions in this population could help to reduce the negative effect of MV on motor development. In the study carried out by Souza et al. (37) it was shown that a therapy protocol based on physical exercise can reduce the time of exposure to mechanical ventilation and oxygen support (38), as well as obtain benefits on the autonomic system (regulation of heart rate and cardiac output), lower rate of complications, increased muscle strength and functional capacity.

Studies such as that of Zhang X, et al. (39), where it is shown that the environmental factors found in the units are a critical factor at the time of neonatal development, with a high probability of presenting different alterations, especially in the behavioral area and in sensitivity; and Mendonça et al. (40) who explain the relationship of ventilatory support with the increase of abnormal movements and atypical development, have a strong clinical implication, to take preventive measures to help reduce the complications that can occur in the neurodevelopment of premature infants. Therefore, the implementation of early care protocols could have a positive impact on preterm infants who are exposed to mechanical ventilation. Valizadeh, et al. (41) demonstrated that, although a passive physical activity program compared to restraint measures in preterm patients does not have a significant impact on neurological development, it does improve motor function of the lower extremities; on the other hand, Hae Yean, et al. (14) concluded that early physical activity programs in the units have a positive impact on the development of low-birth-weight preterm infants in mental and motor functions, achieving significant improvements.

Other research has shown that in addition to improving motor performance, the treatments implemented by physical therapists also bring benefits in bone mineralization, weight gain, better sleep quality and pain reduction (41, 42). Likewise, it has been proven that the techniques used by professionals are safe for patients in critical care units, both high and low risk; however, each case should be evaluated independently, in order to do the best possible good for our premature infants (43).

## 5 Limitations of the study

The primary limitation of this review is the paucity of research exploring the relationship between mechanical ventilation and neonatal neurodevelopment, as well as the complexity of neonatal outcomes and the wide variety of contributing factors, which can make it difficult to draw definitive conclusions. Some studies linked mechanical ventilation to reduced mobility but not to functional outcomes, often using non-standardized outcome measures, which further limited the sample size in the included studies. Additionally, these studies did not distinguish between low birth weight and extremely low birth weight, which would have allowed for a more detailed analysis of this variable and its relationship to mechanical ventilation.

Another limitation is that most studies did not compare outcomes with a group of preterm infants who were not exposed to ventilatory support, making it difficult to confirm the level of risk associated with mechanical ventilation in this population. Additionally, the studies did not account for differences in severity among varying degrees of prematurity, which could influence the reported outcomes.

The lack of uniformity in outcome measures, combined with the fact that the authors did not compare different MV techniques (such as nasal vs. oral intubation, or different models of invasive and non-invasive MV), prevented the possibility of conducting a meta-analysis and highlights a potential area for future research.

## 6 Conclusions

Studies indicate that mechanical ventilation is an independent risk factor for neurodevelopment in low-birth-weight preterm infants. Research indicates that a shorter exposure time and less invasive ventilatory measures can reduce the probability of presenting neurodevelopmental alterations; however, other factors such as ventilation modality and ventilatory parameters are not directly related to the alterations presented by premature infants in the three spheres of neurodevelopment, because they are variables that depend on the health conditions of each premature infant; in addition, the lack of research on these last two factors makes it difficult to conclude their impact on neonates. Therefore, there is a need to continue studying the relationship between ventilatory support and neurological development in a population at risk of presenting alterations during their first years of life, such as low-birth-weight preterm infants.

## Data Availability

The original contributions presented in the study are included in the article/Supplementary Material, further inquiries can be directed to the corresponding author.
